# Practice Variations in Pediatric Hypoglycemia Management: A Survey of Emergency Medicine Physicians

**DOI:** 10.7759/cureus.98399

**Published:** 2025-12-03

**Authors:** Kathryn Keenan, Vincent Calleo, Susan Wojcik, Matthew Thornton

**Affiliations:** 1 Pediatric Emergency Medicine, Upstate Medical University, Syracuse, USA; 2 Toxicology, Upstate Medical University, Syracuse, USA; 3 Emergency Medicine, Upstate Medical University, Syracuse, USA

**Keywords:** dextrose bolus, hypoglycemia management, hypoglycemia threshold, hypoglycemia treatment, pediatric hypoglycemia

## Abstract

Objective

There are several treatment guidelines for the management of hypoglycemia in the pediatric population. The goal of this study was to better understand current practice patterns in the management of pediatric hypoglycemia in the emergency department setting.

Methods

A survey consisting of clinical vignettes was used to gather information on the treatment threshold for both symptomatic and asymptomatic hypoglycemia. The survey also asked about the dose of intravenous dextrose used to correct this problem. This survey was distributed using pediatric and general emergency medicine listservs.

Results

For asymptomatic patients with hypoglycemia, 29.9% (55/184) of respondents stated that they would not typically give a dextrose bolus before trialing oral intake to increase blood glucose levels, regardless of glucose value. For those who had a glucose threshold at which they would give an intravenous dextrose bolus, 29.3% (54/184) indicated that it was at a glucose level less than 50 mg/dL. If the asymptomatic patient had already failed an oral challenge, 39.7% (73/184) of respondents would intervene at a threshold below 60 mg/dL. For patients who had symptoms that could be attributed to hypoglycemia, 95.6% (175/183) would give an intravenous dextrose bolus immediately, as opposed to trialing oral intake first. The most common threshold below which physicians would intervene was 60 mg/dL (36.6%, 67/183). When treating hypoglycemia, 81.0% (149/184) of respondents chose options equivalent to 500 mg/kg of dextrose.

Conclusions

This study illustrates significant practice variation in the management of pediatric hypoglycemia in terms of the blood glucose threshold for intravenous dextrose treatment. The dextrose bolus size had less variation between physicians, with most surveyed following the classically taught 500 mg/kg of dextrose. Under or overtreatment has implications for patient-centered outcomes, including length of stay, and this variation may indicate the need for more studies to better define the risks and benefits of each treatment plan for standardization of care.

## Introduction

Reduced oral intake during a viral illness often leads to hypoglycemia in otherwise healthy children. When hypoglycemia is identified in the emergency department (ED), it is common practice to give an intravenous (IV) bolus of dextrose. The common emergency medicine standard for treating hypoglycemia in children is 500 mg/kg of dextrose [1,2]. This is the equivalent of 5 mL/kg of a 10% dextrose solution and is consistent with the World Health Organization recommendations [3]. Current adult and pediatric emergency medicine recommendations include a range of 200 mg/kg to 1 g/kg of a dextrose bolus [1,2,4,5]. In 2015, the Pediatric Endocrine Society released guidelines recommending a bolus of 200 mg/kg [6].

The recommendation for smaller boluses is in part due to concerns for rebound hypoglycemia [7]. IV dextrose boluses can lead to symptomatic hypoglycemia in certain populations, including patients with long-term malnutrition, such as patients with anorexia nervosa [8]. It is unclear if the rebound hypoglycemia secondary to traditional IV bolus size reaches clinical significance in previously healthy children with hypoglycemia related to a short-term decreased oral intake. A prospective observational study of hypoglycemic adults in the emergency department receiving 50 ml of 50% dextrose solution with serum glucose recorded for 1 hour post-bolus, showed hyperglycemia but no hypoglycemic episodes [9].

In addition to variation in bolus recommendations for hypoglycemia, there are variations in how practitioners define hypoglycemia. In adult patients, hypoglycemia is defined according to Whipple’s triad, which requires low blood glucose, symptoms of hypoglycemia, and resolution of symptoms following glucose intake [10]. This definition can have limited practicality in younger patients who are less able to communicate their symptoms, which leads practitioners to rely more on a specific blood glucose level to define hypoglycemia. The 2015 pediatric endocrinology guidelines recommend evaluating infants and younger children for hypoglycemia when blood glucose measurements are less than 60 mg/dL [6]. The World Health Organization defines hypoglycemia in previously healthy children as less than 45 mg/dL [3]. Others define hypoglycemia as blood glucose less than 47 mg/dL or less than 50 mg/dL [1,2]. The National Board of Medical Examiners, which creates the United States Medical Licensing Examination, has a list of normal reference ranges for laboratory values that includes a reference range of 70 mg/dl - 100 mg/dl for fasting glucose [11]. That is just a reference range, and reference ranges typically refer to 95% of the reference population [12]. 

Given the variations in guidelines, physicians may also look to primary source literature to determine where to draw the treatment line. The goal of treating hypoglycemia is to both resolve acute symptoms and prevent long-term harm. Much of what we know about the long-term effects of hypoglycemia comes from neonates. A 2019 meta-analysis of neurodevelopmental outcomes in children exposed to neonatal hypoglycemia found increased visual-motor impairment, executive dysfunction, and general cognitive impairment in early childhood, as well as literacy and numeracy problems in later childhood. The studies included in the meta-analysis had different definitions of hypoglycemia from <20 mg/dl to <47 mg/dL [13]. An MRI study of neonates exposed to glucose levels <47 mg/dL showed that those children had statistically significantly smaller caudate, thalamus, and subcortical grey matter volumes in later childhood [14]. The duration of hypoglycemia seems to matter as well. A follow-up study of neonates exposed to hypoglycemia showed worse two-year-old adaptability scores for infants exposed longer to hypoglycemia or to more repeated episodes of hypoglycemia. This adaptability score included components of fine-motor coordination for objects and scenes, hand-eye coordination, problem solving, and application tools [15]. Although there is data supporting adverse long-term neurocognitive effects at these levels in neonates, it is unclear how well that translates to older children.

Given the wide variety of recommendations, we sought to better understand what the current hypoglycemia management practices are, both in terms of serum glucose level treatment threshold and in terms of dextrose bolus size, for pediatric patients in emergency departments in the United States.

## Materials and methods

This was a multiple-choice survey study comprising four clinical vignettes to determine glucose treatment threshold, one question on dextrose bolus size used for hypoglycemia, one question on whether their institution has a hypoglycemia protocol, and five questions on the practitioners themselves and the patient population they serve. 

Four clinical vignettes were created, each with the presentation of a previously healthy 18-month-old with a history of decreased oral intake in the setting of an acute illness. Two of the vignettes presented a child with low glucose, but without obvious symptoms of hypoglycemia. One of the vignettes was set before a glucose challenge was attempted, and one after the patient failed an oral glucose challenge. We then asked if there was a glucose level below which the physician would typically give that child an IV dextrose bolus. The other two vignettes asked the same question, but presented a child who clinically showed symptoms that could be attributed to hypoglycemia.

Vignettes were evaluated by a panel of pediatric emergency medicine physicians for pilot testing. Afterwards, we spoke to the physicians to gather feedback to ensure realism and accuracy of intended interpretation.

The survey was distributed to the Pediatric Emergency Medicine fellowship director listserv, the Emergency Medicine program director listserv, and the Brown Pediatric Emergency Medicine national listserv utilizing a public link to REDCap® (Vanderbilt University, Nashville, USA). Survey responses were collected over the next two months, from December 2022 to January 2023.

This study was approved under Institutional Review Board (IRB) exempt status by our institution. Data were summarized using descriptive statistics.

## Results

A total of 191 responses were received. The total number of people sent the survey is unknown. Ten of the responses received were incomplete. Each of the questions were analyzed individually for all the responses given, even if the respondent didn’t answer all questions.

Most respondents were attending physicians (82.9%, 150/181) and the rest were pediatric emergency medicine fellows. The primary practice setting of 85.2% (155/182) of respondents cared for pediatric patients only. One-third (62/186) of physicians had an institutional protocol for management of hypoglycemia outside of the neonatal age group. 

**Figure 1:**
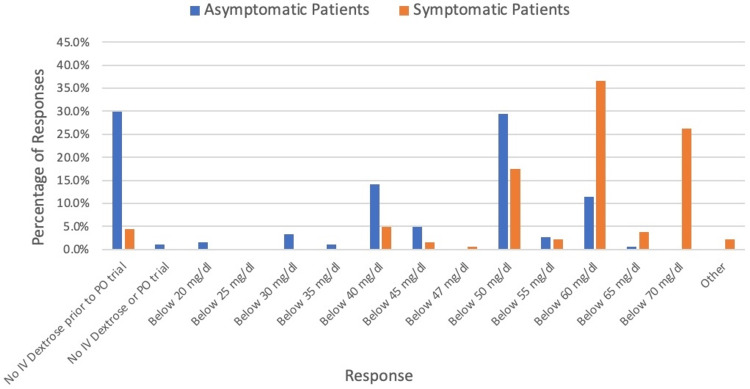
IV Dextrose Bolus Treatment Threshold for Hypoglycemia Patients Prior to PO Trial IV: intravenous; PO: per oral.

**Figure 2:**
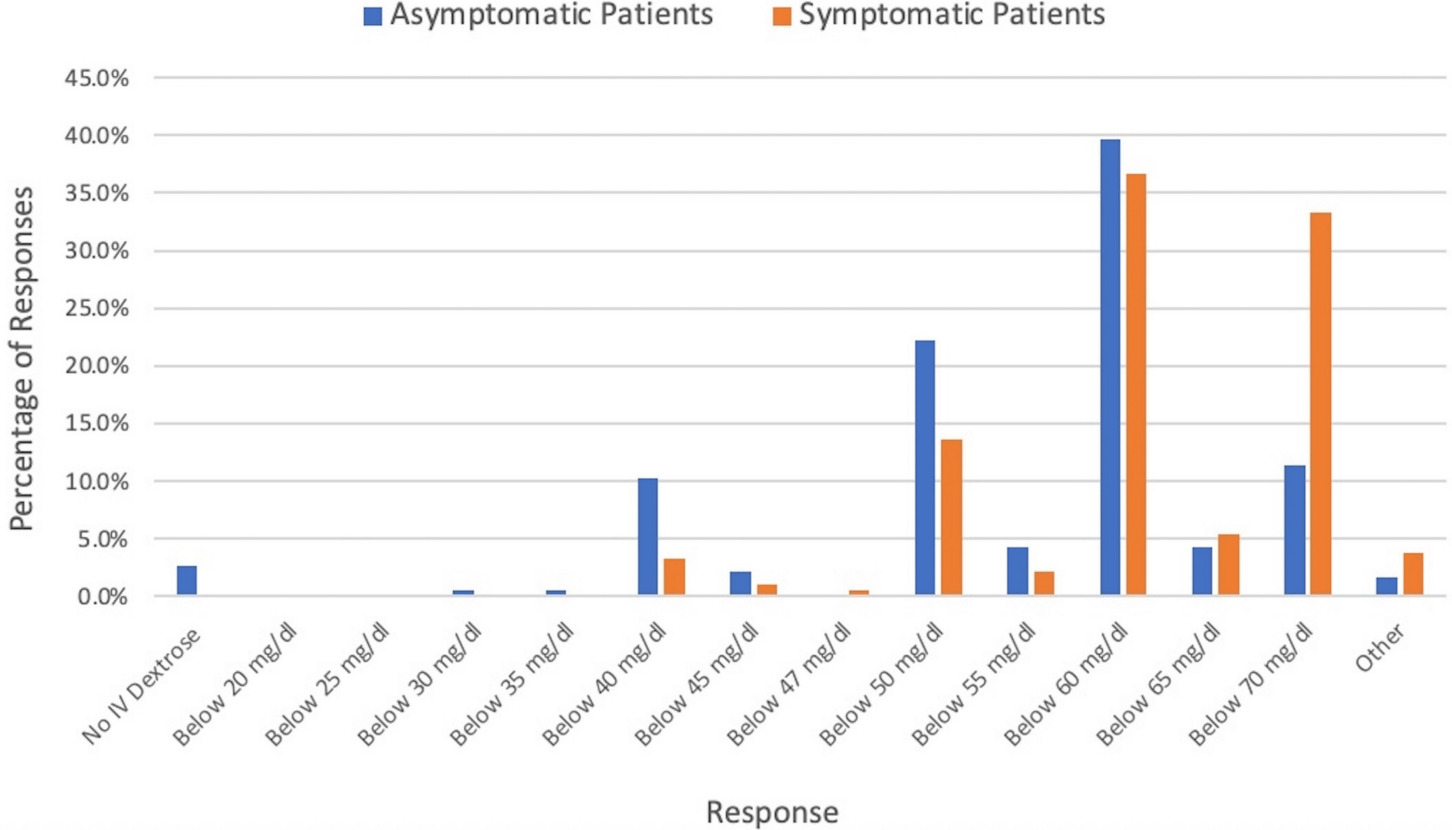
IV Dextrose Bolus Treatment Threshold for Hypoglycemia Patients After Failed PO Trial IV: intravenous; PO: per oral.

For asymptomatic patients with hypoglycemia, 29.9% (55/184)) of respondents stated that they would not typically give a dextrose bolus before trialing per oral (PO) regardless of glucose value. For those who had a glucose threshold at which they would give an IV dextrose bolus, 29.3% (54/184) indicated that was at a glucose less than 50 mg/dL (Figure 1). If the asymptomatic patient had already failed a PO trial 39.7% (73/184) of respondents would intervene at a threshold of below 60 mg/dL (Figure 2).

For patients who had symptoms that could be attributed to hypoglycemia, 95.6% (175/183) would give an IV dextrose bolus immediately as opposed to trialing PO first. The most common threshold below which the physicians would intervene was 60 mg/dL (36.6%, 67/183) (Figure 1).

Regarding the dextrose bolus choice, 73.9% (136/184) of respondents chose a bolus of 5 mL/kg of D10. A total of 81.0% (149/184) of respondents chose options equivalent to 500 mg/kg of dextrose and consistent with “the rule of 50s”. Only 13.0% (24/184) of respondents chose boluses that would provide less than 500 mg/kg of dextrose. 

## Discussion

The surveyed physicians were split on whether there was a glucose value below which they would automatically treat before trialing PO in an asymptomatic patient. Those who would wait may reflect the Whipple’s triad approach to hypoglycemia. Those who reference a specific value may reflect an acknowledgement of the uncertainty of recognizing hypoglycemic symptomatology in younger pediatric patients.

In patients who displayed symptoms that could be related to hypoglycemia, most physicians had a glucose threshold at which they would intervene rather than waiting for a PO trial. A threshold of less than 60 mg/dL was the most common treatment line for all the example patients, except for the asymptomatic patient who had not yet trialed PO. In that patient scenario, the most common treatment line was 50 mg/dL. Although we do not have evidence related to long-term harms of hypoglycemia in previously healthy patients coming to the ED with an acute illness, extrapolation from neonatal hypoglycemia literature suggests this is a reasonable treatment threshold [13,14]. Although those were the most common treatment thresholds for those clinical scenarios, there was still significant variation. For asymptomatic patients after failed PO challenge for those who had a treatment threshold, answers ranged from below 30 mg/dl to below 70 mg/dl. For those with symptoms that could be attributed to hypoglycemia, both in the case of no PO trial yet and in the case of a failed PO trial, glucose thresholds for treatment ranged from below 40 mg/dl to below 70 mg/dl.

Although the Pediatric Endocrinology Society guidelines reference 60 mg/dl as the level for hypoglycemia, many respondents indicated they would treat for hypoglycemia at higher levels. This may reflect the different priorities and clinical scenarios in which different specialties work. Emergency medicine practitioners often have a priority of ruling out serious pathology and may be more likely to treat to figure out if the presenting symptomatology could be related to hypoglycemia or if other causes need to be considered. 

For the size of the dextrose bolus, there was significantly less variation in practice. Most practitioners surveyed follow the classically taught bolus equivalent of 500 mg/kg of dextrose, though some are using smaller bolus doses that are in line with the Pediatric Endocrine Society recommendations. There may be benefits to the smaller bolus size; however, more information is needed pertaining to the previously healthy child with acute decreased oral intake. 

There are multiple weaknesses of this survey study. One of the biggest limitations is that we cannot quantify the total number of people who received the survey, so we cannot obtain a true response rate, and the results may not accurately represent the larger population. We also do not know which area of the country we obtained the responses from, which also makes it more difficult to determine whether this study is widely applicable. As this is a survey study, we have reported clinical practice, and cannot verify objective clinical practice. We did not receive enough responses from general ED providers to differentiate their practice patterns from those who specialize specifically in pediatric emergency medicine. Additionally, we did not have any responses from non-physicians, so we are not able to determine if there are variations in practice related to provider type.

## Conclusions

This study suggests that there is significant practice variation in the management of hypoglycemia in pediatric patients, particularly related to the glucose threshold at which patients are treated. If children are treated for hypoglycemia at a glucose level that is not clinically relevant, that could lead to unnecessary IV placement or a prolonged stay in the ED for treatment without clinical benefit. If left at too low a glucose level without intervention, that could lead to a prolonged stay in the ED or hospital due to failure to intervene to help the patient feel better and return to the clinical baseline. 

The dextrose bolus size had less variation between physicians, with most surveyed following the classically taught 500 mg/kg of dextrose. There is a theoretical risk of rebound hypoglycemia if the bolus of dextrose is too large, which could prolong the patient's stay. However, an insufficient dextrose bolus may not adequately raise the blood sugar. As of now, more studies are needed to determine if any one treatment strategy regarding dextrose bolus or glucose level treatment threshold is superior to others. This would hopefully allow pediatric emergency medicine teaching and pediatric endocrinology society guidelines to more closely align for the standardization of care. 
